# Terahertz plasmon and surface-plasmon modes in hollow nanospheres

**DOI:** 10.1186/1556-276X-7-578

**Published:** 2012-10-23

**Authors:** Yiming Xiao, Wen Xu, Yaya Zhang, Jiaguang Hu

**Affiliations:** 1 Department of Physics, Yunnan University, Kunming, 650091, China; 2 Key Laboratory of Materials Physics, Institute of Solid State Physics, Chinese Academy of Sciences, Hefei, 230031, China; 3 Department of Math and Physics, Wenshan University, Wenshan, 663000, China

**Keywords:** Hollow nanosphere, Electronic subband structure, Collective excitation modes, Terahertz radiation

## Abstract

We present a theoretical study of the electronic subband structure and collective electronic excitation associated with plasmon and surface plasmon modes in metal-based hollow nanosphere. The dependence of the electronic subband energy on the sample parameters of the hollow nanosphere is examined. We find that the subband states with different quantum numbers *l* degenerate roughly when the outer radius of the sphere is *r*_2_ ≥ 100 nm. In this case, the energy spectrum of a sphere is mainly determined by quantum number *n*. Moreover, the plasmon and surface plasmon excitations can be achieved mainly via inter-subband transitions from occupied subbands to unoccupied subbands. We examine the dependence of the plasmon and surface-plasmon frequencies on the shell thickness *d* and the outer radius *r*_2_ of the sphere using the standard random-phase approximation. We find that when a four-state model is employed for calculations, four branches of the plasmon and surface plasmon oscillations with terahertz frequencies can be observed, respectively.

## Background

In recent years, there has been a great interest in the investigation of metal-based hollow nanostructures because of their unique characteristics such as low density, large specific area, mechanical and thermal stability, and surface permeability. These advanced materials have been widely applied in catalysis [1], drug delivery [2,3], food and cosmetic industries [4], fuel cell [5,6], biotechnology [7], lubricant [8], sensing [9], photonic devices [10], micro/nanoreactors [11], etc. In particular, metal-based hollow nanospheres [12] can be realized via using polystyrene (PS) latex particles as templates [13]. Such structures have intriguing features of surface plasmon resonance [14]. The collective oscillations of the conducting electrons in response to optical excitation, such as plasmon and surface plasmon excitations, affect strongly the optical properties of metal hollow nanospheres. At present, it has become possible to fabricate metal hollow nanosphere structures in which the radius and shell thickness of the sphere can be controlled artificially. Such structures have been widely applied to realize terahertz (10^12^ Hz or THz) plasmonic devices [15]. Hence, it is of great importance and significance to study the electronic subband structure and corresponding collective electronic excitations from these advanced nanomaterial systems. In conjunction with recent experimental achievement in the field, in this article, we would like to develop a simple theoretical approach to study the electronic subband structure and plasmon and surface-plasmon modes in a hollow nanosphere. The aim of this study is to examine how sample parameters affect the electronic subband energy and the plasmon and the surface-plasmon modes in the device systems.

## Methods

### Theoretical approach

#### Electronic subband structure

In this study, we consider an air/metal-shell/air-based hollow nanosphere structure. The inner radius of the structure is *r*_1_, the outer radius or the diameter of the sphere is *r*_2_, and the metal shell thickness is *d *=* r*_2_ −* r*_1_. Such structure can be realized experimentally by selectively removing the hard spherical core (e.g., PS latex particles) in the fabrication process [16]. For a case where the electrons in the metal shell are not tunneling or hopping into the core and outside air, the confining potential for electrons in the structure can be modeled simply as

(1) $$V(r)=\left\{\begin{array}{cc} 0, & r_{1}\leq r\leq r_{2};\\ \infty , & \text{otherwise}. \end{array}\right)$$

Thus, the corresponding Schrödinger equation takes a form

(2) $$[P^{2}/2\mu +V(r)]\psi_{N}(R)=E_{N}\psi_{N}(R).$$

Here, **P **= (*p*_*x*_,*p*_*y*_,*p*_*z*_) is the momentum operator, *μ* is the effective mass for an electron in the structure, **R **= (*x*,*y*,*z*) = (*r*,*θ*,*ϕ*), and *N* stands for all quantum numbers. The solution of Equation (2) is $\psi_{N}(r,\theta ,\phi )=R_{\text{nl}}(r)Y_{l}^{m}(\theta )e^{\text{im}\phi }$, where *N *= (*nlm*),

(3) $$\begin{array}{l} Y_{l}^{m}(\theta )={\left(-1\right)}^{(m+|m|)/2}{\left[\frac{(2l+1)(l-|m|)!}{4\Pi (l+|m|)!}\right]}^{1/2}\\ \;\;\times P_{l}^{\left|m\right|}(\text{cos}\theta ),\; \end{array}$$

and *R*_*nl*_(*r*) is determined by

(4) $$\begin{array}{l} \frac{1}{r^{2}}\frac{\partial }{\mathrm{\partial r}}\left(r^{2},\frac{\partial R_{\text{nl}}(r)}{\partial r}\right)-\frac{l(l+1)}{r^{2}}R_{\text{nl}}(r)=\\ \;\;-\frac{2\mu }{\hbar^{2}}[E_{N}-V(r)]R_{\text{nl}}(r).\; \end{array}$$

Here, *l *= 0,1,2,⋯ is the angular momentum quantum number, *m *=* l*,*l*−1,⋯,−*l* is the magnetic quantum number, $P_{l}^{m}(x)$ is the associated Legendre function, and *l* must be a positive integer in the range *l *≥ |*m*|. Letting *E*_*N *_=* ℏ*^2^*k*^2^/2*μ*, *x *=* kr*, and $R_{\text{nl}}(r)=\sqrt{\Pi /2x}\;y(x)$, the radial equation, Equation (4), for *r*_1_ ≤* r *≤* r*_2_ becomes a Bessel equation with a general solution: $y(x)=N_{\text{nl}}[C_{1}J_{l+1/2}(x)+C_{2}J_{-l-1/2}(x)]$, where *J*_*α*_(*x*) is a Bessel function and $N_{\text{nl}}$ is a normalization factor. Considering the boundary conditions: *R*(*r*_1_) = 0 and *R*(*r*_2_) = 0, we have

(5) $$J_{l+1/2}(kr_{2})J_{-l-1/2}(kr_{1})-J_{l+1/2}(kr_{1})J_{-l-1/2}(kr_{2})=0,$$

which is applied to determine the energy spectrum of the sample structure. Thus, the electron wave function becomes

$$\psi_{N}(r,\theta ,\phi )={\left(-1\right)}^{(m+|m|)/2}{\left[\frac{(2l+1)(l-|m|)!}{4\Pi (l+|m|)!}\right]}^{1/2}e^{\text{im}\phi }$$

(6) $$\times P_{l}^{\left|m\right|}(\text{cos}\theta )N_{\text{nl}}\sqrt{\Pi /2\text{kr}}[C_{1}J_{l+1/2}(\text{kr})+C_{2}J_{-l-1/2}(\text{kr})].$$

For case of *l *= 0, we obtain *E*_*n*0_ =* ℏ*^2^*Π*^2^*n*^2^/2*μd*^2^ with *n *= 1,2,⋯. The radial eigenfunction is

(7) $$R_{n0}(r)=\sqrt{\frac{2\overset{2}{\text{cos}}(kr_{1})}{d}}\left[\frac{\text{sin}(\text{kr})}{r},-,\frac{\text{tan}(kr_{1})\text{cos}(\text{kr})}{r}\right],$$

where *k *= *nΠ*/*d*.

For *l *= 1, we have

(8) $$\text{tan}(\text{kd})=\frac{\text{kd}}{1+k^{2}r_{1}r_{2}},$$

and the radial eigenfunction becomes

(9) $$\begin{array}{l} R_{n1}(r)=\frac{N_{n1}}{r}\left(\left[\frac{\text{sin}(\text{kr})}{\text{kr}},-,\text{cos},(,\text{kr},)\right]\right)-\frac{\text{tan}(kr_{1})-kr_{1}}{kr_{1}\text{tan}(kr_{1})+1}\\ \;\;\times \left(\left[\text{sin},(,\text{kr},),+,\frac{\text{cos}(\text{kr})}{\text{kr}}\right]\right). \end{array}$$

For *l *= 2, we get

(10) $$\text{tan}(\text{kd})=\frac{(9+3k^{2}r_{1}r_{2})\text{kd}}{k^{2}(k^{2}r_{1}^{2}r_{2}^{2}-3r_{1}^{2}-3r_{2}^{2}+9r_{1}r_{2})+9}.$$

E_*n*1_ and E_*n*2_ are determined numerically via solving respectively Equations (8) and (10).

#### Electron-electron interaction

The matrix element for the bare electron-electron (e-e) interaction can be obtained by applying the electron wave function to the interaction Hamiltonian induced by the Coulomb potential [17], which reads

(11) $$\begin{array}{l} V_{N_{1}^{\prime }N_{1}N^{\prime }N}=\frac{e^{2}}{\kappa }\int \int \psi_{N_{1}^{\prime }}^{*}(R_{1},\theta_{1},\phi_{1})\psi_{N_{1}}(R_{1},\theta_{1},\phi_{1})\\ \;\;\;\times \frac{1}{\left|R_{1}-R_{2}\right|}\psi_{N^{\prime }}^{*}(R_{2},\theta_{2},\phi_{2})\psi_{N}(R_{2},\theta_{2},\phi_{2}),\; \end{array}$$

with *κ *being the high-frequency dielectric constant of the shell material. It can be simplified as

(12) $$\begin{array}{lll} V_{N_{1}^{\prime }N_{1}N^{\prime }N}= & \underset{k}{\sum }c^{k}(N,N^{\prime })c^{k}(N_{1}^{\prime },N_{1})R^{k}(NN_{1},N^{\prime }N_{1}^{\prime })\; & \;\\ & \times \delta_{m_{N}+m_{N_{1}},m_{N^{\prime }}+m_{N_{1}^{\prime }}}.\; \end{array}$$

Here, ${\text{c}}^{k}$ and ${\text{R}}^{k}$ are, respectively,

(13) $$\begin{array}{l} \;c^{k}(N,N^{\prime })={\left(-1\right)}^{m_{N}-m_{N^{\prime }}}c^{k}(N^{\prime },N)=\sqrt{\frac{2}{2k+1}}\\ \times \overset{\Pi }{\underset{0}{\int }}T(k,m_{N}-m_{N^{\prime }})T(l_{N},m_{N})T(l_{N^{\prime }},m_{N^{\prime }})\text{sin}\theta d\theta , \end{array}$$

where $T(l,m)=\sqrt{2\Pi }Y_{l}^{m}(\theta )$ as shown in Equation (3), and

(14) $$\begin{array}{lll} R^{k}(NN_{1},N^{\prime }N_{1}^{\prime })= & \frac{e^{2}}{\kappa }\int \int \frac{R_{<}^{k}}{R_{>}^{k+1}}R_{N_{1}^{\prime }}(R_{1})R_{N_{1}}(R_{1})\; & \;\\ & \times R_{N^{\prime }}(R_{2})R_{N}(R_{2})R_{1}^{2}R_{2}^{2}dR_{1}dR_{2},\; \end{array}$$

where *R*_<_ (*R*_>_) is the smaller (bigger) value of {*R*_1_,*R*_2_}. In order that *c*^*k*^ can have a non-zero value, k must satisfy the conditions

(15) $$\begin{array}{l} k+l_{N}+l_{N^{\prime }}=2g(g\text{is an integer})\\ \;|l_{N}-l_{N^{\prime }}|\leq k\leq |l_{N}+l_{N^{\prime }}|. \end{array}$$

Table 1 gives the values of $c^{k}$ for *s* and *p* electrons in case of *m *= 0.

**Table 1:** $c^{k}(N^{\prime },N)$

***l***_***N***_	$l_{N^{\prime }}$	***m***_***N***_	$m_{N^{\prime }}$	***c***^**0**^**(*N***^***′***^**,*N*)**	***c***^**1**^**(*N***^***′***^**,*N*)**	***c***^**2**^**(*N***^***′***^**,*N*)**
*s*	*s*	0	0	1		
*p*	*p*	0	0	1		4
*s*	*p*	0	0		1	

*l*_*N*_ and *m*_*N*_ are angular momentum quantum number and magnetic quantum number for a quantum state *N*, respectively, whereas $l_{N^{\prime }}$ and $m_{N^{\prime }}$ for a quantum state *N*^*′*^. *c*^0^(*N*^*′*^,*N*), *c*^1^(*N*^*′*^,*N*), and *c*^2^(*N*^*′*^,*N*) are angle factors for k=0,1,2 defined by Equations (13) and (15).

#### Plasmon and surface-plasmon modes

From electron energy spectrum obtained from the solution of the Schrödinger equation, we can derive the retarded and advanced Green’s function for electrons. Applying these Green’s functions and *V*_*αβ*_ with *β *=*N*^*′*^*N* to the diagrammatic techniques to derive effective e-e interaction under the random phase approximation, the element of the dielectric function matrix is obtained as [17,18]

(16) $$\varepsilon_{\alpha \beta }(\Omega )=\delta_{\alpha ,\beta }-V_{\alpha \beta }\pi_{\beta }(\Omega ),$$

where

(17) $$\pi_{N^{\prime }N}(\Omega )=\frac{g_{s}[f(E_{N^{\prime }})-f(E_{N})]}{\hbar \Omega +E_{N^{\prime }}-E_{N}+i\delta }$$

is the pair bubble (or density-density correlation function) in the absence of e-e coupling with *g*_*s *_= 2, counting for spin degeneracy, and *f*(*E*_*N*_) = ${\left[1+e^{(E_{N}-E_{F})/k_{B}T}\right]}^{-1}$ being the Fermi-Dirac function.

**Figure 1:**
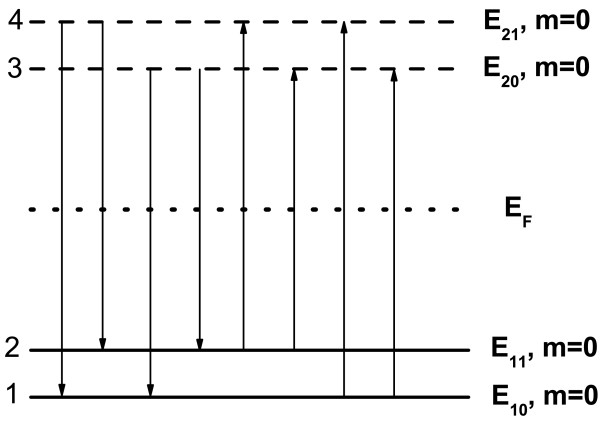
**The four-state model for electronic transitions in a hollow nanosphere.** E_10_ and E_11_ are occupied states, and E_20_ and E_21_ are unoccupied states. *E*_F_ is the Fermi energy. Here, we consider the electronic states in the case of *m *= 0. The possible transition channels are indicated.

In a hollow nanosphere system described by quantum number *N *= (*nlm*), the electronic subband energy depends only on *n* and *l* quantum numbers, namely *E*_*N *_=* E*_*nl*_. In this study, we consider that *n *= 1 states with many *l* and *m* numbers are occupied, and *n *= 2 states with any *l* and *m* numbers are unoccupied. For simplicity, we take a four-state model (FSM) to calculate the dielectric function matrix. We consider that two lowest electronic states for *n *= 1, E_10_, and E_11_ are occupied, and two lowest electronic states for *n *= 2, E_20_, and E_21_ are unoccupied, as shown in Figure 1. Because the electronic subband energy in a hollow sphere does not depend on the quantum number *m*, we take *m *= 0 in the calculations. On the basis that all electronic states in a hollow nanosphere are quantized, intra-subband transitions do not contribute to dielectric function. Moreover, the transitions within the occupied and within the unoccupied states do not contribute to the dielectric function as well. Thus, as shown in Figure 1, there are eight possible transition channels induced by inter-subband transitions from occupied (unoccupied) states to unoccupied (occupied) states in this FSM. Setting the electronic state index as 1 = (100), 2 = (110), 3 = (200), and 4 = (210), the dielectric function of a hollow nanosphere in the FSM is a 16×16 matrix and can be obtained from Equation (16). The determinant of the dielectric function matrix then is

(18) $$\begin{array}{lll} |\varepsilon (\Omega )|= & [(1+a_{3131}+a_{3113})(1+a_{4242}+a_{4224})\; & \;\\ & -(a_{3142}+a_{3124})(a_{4231}+a_{4213})]\; & \;\\ & \times [(1+a_{4141}+a_{4114})(1+a_{3232}+a_{3223})\; & \;\\ & -(a_{3241}+a_{3214})(a_{4132}+a_{4123})],\; \end{array}$$

with *a*_*αβ *_= −*V*_*αβ*_*π*_*β*_(*Ω*). The plasmon and surface-plasmon modes are determined by Re|*ε*(*Ω*)|→0 and Re|*ε*(*Ω*)|→−1, respectively. In this study, we employ a matrix to present the dielectric function in a multi-energy level system such as a hollow nanosphere structure. Such an approach was applied to study the plasmon excitations in semiconductor-based two-dimensional electron gas systems [19] and Rashba spintronic systems [18]. We note that in the present study, we consider a simple model to calculate the electronic subband structure of a hollow nanosphere. The effect of the spin-orbit interaction in the system is not included.

## Results and discussion

**Figure 2:**
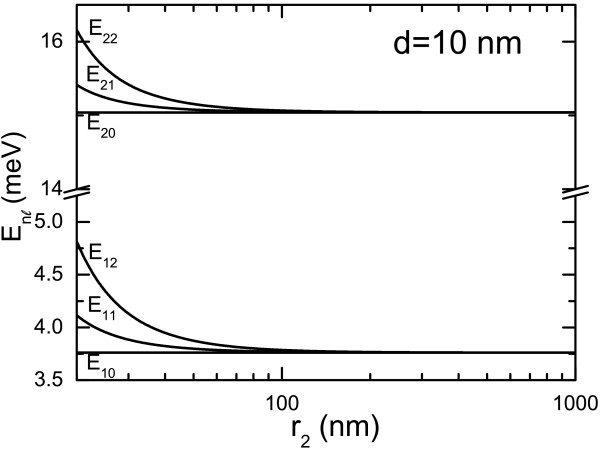
**The subband energies vary with *r***_**2**_** at a fixed* d*.** Electronic subband energy, E1_*l *_and E2_*l*_for *l* = 0, 1, and 2, as a function of outer radius *r*_2_ of hollow nanosphere at a fixed shell thickness *d* = 10 nm.

**Figure 3:**
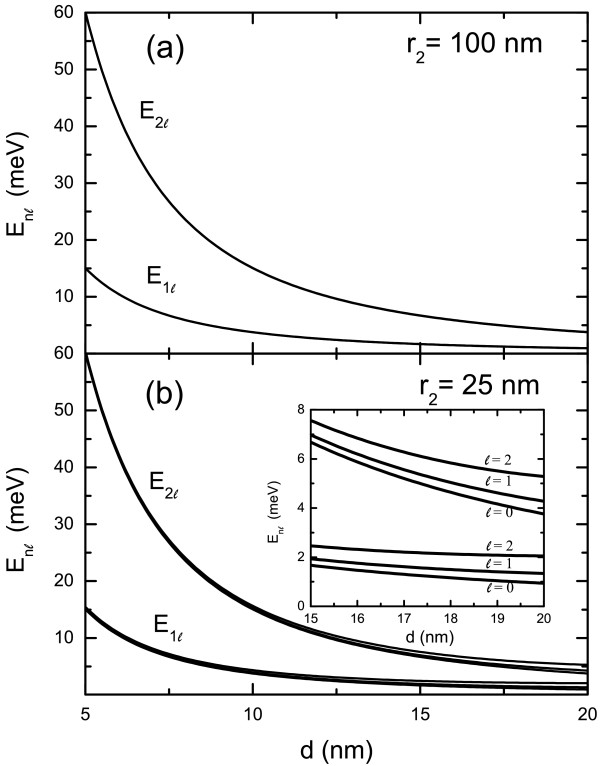
**The subband energies vary with *d *at fixed *r***_**2**_**.** Electronic subband energy, E1_*l *_and E2_*l*_for *l* = 0, 1, and 2, as a function shell thickness *d* of hollow nanosphere for outer radius *r*_2_ = 100 nm in **(a)** and *r*_2_ = 25 nm in **(b)**. The inset in **(b)** shows the energy difference in different *l* states.

In numerical calculations, we take the effective mass for an electron to be about the rest electron mass, i.e., *μ *≃ 0.99*m*_*e*_, and the high-frequency dielectric constant *κ *= 1.53 for gold shell [20-22]. In Figure 2, we show the electronic subband energy for E1_*l*_ and E2_*l*_(*l *= 0, 1, and 2) as a function of outer radius *r*_2_ of the hollow nanosphere with a fixed shell thickness *d *= 10 nm. We see that the energy levels with different *l* quantum numbers roughly degenerate when *r*_2_ > 100 nm. In such a case, the subband energy depends very little on *r*_2_and *E*_*nl *_≃* E*_*n*0_ =* ℏ*^2^*Π*^2^*n*^2^/2*μd*^2^. In Figure 3a, the electronic subband energies for E1_*l *_and E2_*l*_(*l *= 0, 1, and 2) are shown functions of shell thickness *d* at a fixed outer radius *r*_2_ = 100 nm of the hollow nanosphere. The results for different *l* states coincide roughly. The subband energy decreases with increasing shell thickness as *E*_*nl *_≃* E*_*n*0_∼*d*^−2^. In Figure 3b, the electronic subband energies for E1_*l*_and E2_*l*_(*l *= 0, 1, and 2) are shown functions of shell thickness *d* for a fixed outer radius *r*_2_ = 25 nm of the hollow nanosphere. The subband energies degenerate roughly at small shell thickness and show difference with increasing shell thickness *d* as shown in the inset in Figure 3b. We know that the energy for the *n*^*th*^ subband at *l *= 0 is determined only by the shell thickness *d *=* r*_2_ −* r*_1_ of a hollow nanosphere. The results shown in Figures 2 and 3 indicate that different *l* states degenerate at a fixed *n* quantum number when *r*_2_ > 100 nm. This feature is mainly induced by the symmetry of the confining potential for electrons, given as Equation (1). However, when *r*_2_ is relatively small (see Figure 2 and Figure 3b), the electronic subband energy depends on quantum number *l* for a fixed quantum number *n* and *E*_*nl*_>*E*_*n*0_ (here *l *> 0). This suggests that the stronger quantum effect can be achieved in smaller sample structures. Such an effect can be understood by the fact that when *r*_2_→*∞*, the energies determined by Equations (8) and (10) approach *E*_*nl *_→* E*_*n*0_ =* ℏ*^2^*Π*^2^*n*^2^/2*μd*^2^ and when *r*_2_ takes a finite value *E*_*nl *_>* E*_*n*0_.

**Figure 4:**
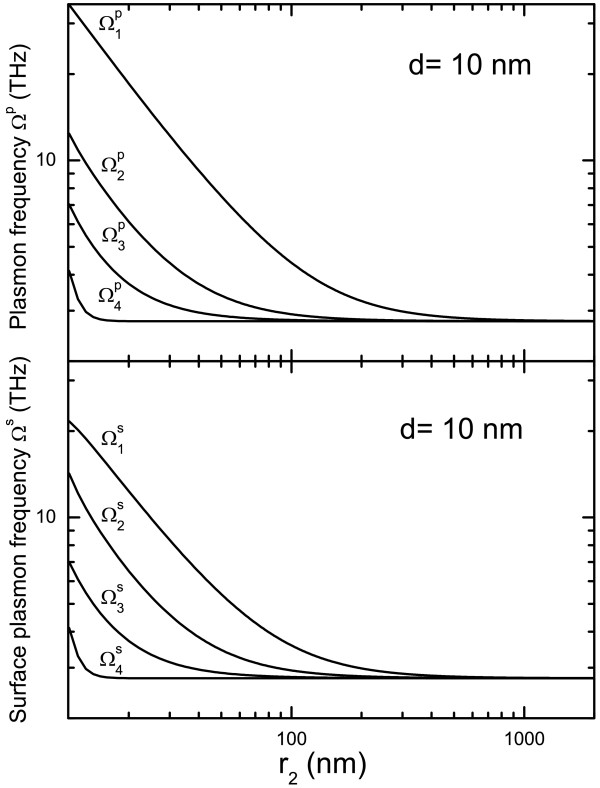
**Dependence of plasmon and surface-plasmon frequencies on outer radius* r***_***2***_** at a fixed shell thickness *d *= 10 nm.**

In Figure 4, the plasmon and surface-plasmon frequencies of hollow nanosphere are shown as a function of outer radius *r*_2_ at a fixed shell thickness *d*. Using the FSM, there are four modes for both plasmon and surface-plasmon excitation from a hollow nanosphere. We see that (1) the plasmon and surface-plasmon frequencies decrease with increasing *r*_2_ when *r*_2_ < 200 nm. When *r*_2_ > 200 nm, the plasmon and surface-plasmon frequencies approach approximately to the energy-gap between *E*_20_ and *E*_10_; (2) the frequencies of all these modes are in the THz regime; (3) the plasmon frequency $\Omega_{1}^{p}≃\sqrt{2}\Omega_{1}^{s}$ with $\Omega_{1}^{s}$ being a surface-plasmon frequency. This is the primary relationship between plasmon and surface-plasmon modes; (4) the surface-plasmon frequency $\Omega_{2}^{s}$ is slightly higher than plasmon frequency $\Omega_{2}^{p}$; and (5) $\Omega_{4}^{p}≃\Omega_{4}^{s}$ and $\Omega_{3}^{p}≃\Omega_{3}^{s}$.

**Figure 5:**
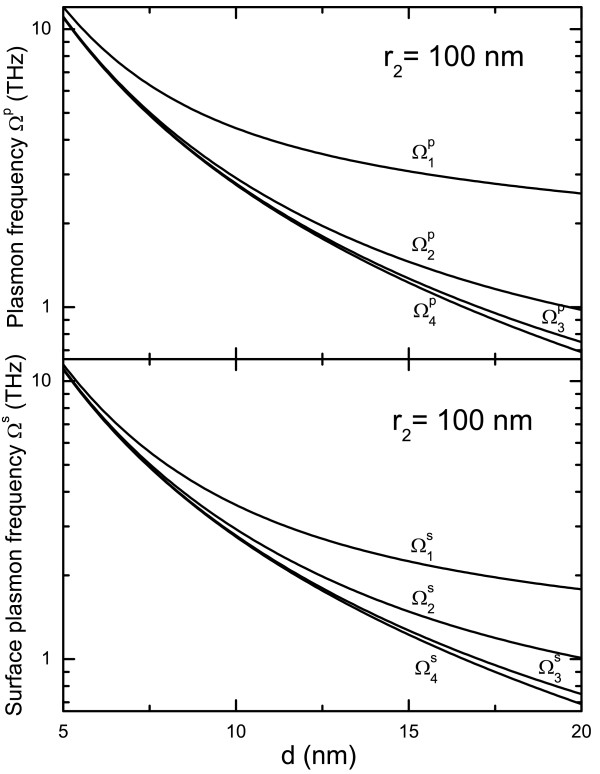
**Dependence of plasmon and surface-plasmon frequencies on the shell thickness *d* at a fixed outer radius* r***_**2**_*** = 100 nm.***

In Figure 5, the plasmon and surface-plasmon frequencies are shown as functions of shell thickness *d* at a fixed *r*_2_. The plasmon and surface-plasmon frequencies decrease with increasing shell thickness. The frequency difference between different excitation modes gets wider with increasing *d*.

It should be noted that when *r*_2_ > 100 nm and *d *∼ 10 nm, *E*_*nl *_≃* E*_*n*0_, and plasmon and surface-plasmon frequencies in a hollow nanosphere are determined mainly by transition events between *E*_2*l *_and *E*_1*l*_. This implies that although only four electronic subbands are included within current calculations, the obtained results should be very much similar to the case where more electronic states are considered when *r*_2_ > 100 nm and *d *∼ 10 nm. The results obtained from this study indicate that the electronic subband energy and the plasmon and surface-plasmon modes in hollow nanospheres are determined mainly by sample parameters such as the diameter of the sphere *r*_2_ and the shell thickness *d*. When *r*_2_ > 100 nm, the energy levels depend very weakly on inner or outer radius (i.e., *r*_1_ or *r*_2_) at a fixed *d*. Thus, the shell thickness affects more strongly the electronic subband energies in a hollow nanosphere. We find that when *d *∼ 10 nm and *r*_2_ ≥ 100 nm, the energy spacing between E2_*l *_and E1_*l*_ states is about 10 meV or about 2.4 THz. The frequencies of plasmon and surface-plasmon modes in the structure are also in the THz bandwidth. The plasmon and surface-plasmon modes depend sensitively on the geometrical parameters such as the outer radius *r*_2_ and shell thickness *d*. These effects imply that metal-based hollow nanosphere structures can be applied as THz materials or devices in which THz optical absorption and excitation can be achieved via inter-subband electronic transitions. It is known that THz technology is of great potential to impact many interdisciplinary fields such as telecommunication, biological science, pharmaceutical technology, anti-terrorist, etc. [23]. The application of nanostructure in THz technology has become a fast growing field of research in recent years. The theoretical findings from this work confirm that hollow gold-nanosphere structures are indeed the THz plasmonic materials which can be applied as frequency-tunable THz optoelectronic devices.

## Conclusions

In this study, we have examined theoretically the electronic subband structure and the plasmon and surface-plasmon modes of hollow nanosphere structures. We have found that when the diameter of the sphere *r*_2_ > 100 nm and the shell thickness *d *∼ 10 nm, the energy levels for different *l* states roughly degenerate. In such a case, the electronic subband energy, *E*_*nl *_≃* E*_*n*0_ =* ℏ*^2^*Π*^2^*n*^2^/2*μd*^2^, does not depend on *r*_2_. When *r*_2_ < 200 nm, the plasmon and surface-plasmon modes induced by different electronic transition channels have significantly different frequencies. When *r*_2_ > 200 nm, the plasmon and surface-plasmon frequencies approach roughly to *Ω*^*p *^∼* Ω*^*s *^∼ (*E*_20_ −* E*_10_)/*ℏ*, which depend largely on *d* and depend very little on *r*_2_.

It should be noted that at present, little research work has been carried out to look into the electronic subband structure of the hollow nanosphere structures using more powerful theoretical tools such as the first principle calculations which require large scale numerical computations and are CPU-consuming. The simple analytical results obtained from this study can be applied further to study the electronic and optoelectronic properties of the hollow nanosphere structures. We have found that the plasmon and surface-plasmon excitations can be achieved via inter-subband electronic transition channels in the hollow nanospheres. In particular, we have demonstrated that in metal hollow nanospheres, the energy difference between E1_*l*_ and E2_*l*_ states, and the plasmon and surface-plasmon frequencies are all in the THz bandwidth. This can lead to an application of metal hollow nanosphere structures in THz optics and optoelectronics.

## Competing interests

The authors declare that they have no competing interests.

## Author’s contributions

WX proposed and supervised the research work. YX carried out the analytical and numerical calculations. YZ and JH participated in the discussions and analyzes of the obtained results. All authors read and approved the final manuscript.

## Author’s information

WX is the distinguished professor at Yunnan University and research professor at the Institute of Solid State Physics, Chinese Academy of Sciences. YX and YZ are post-graduate students at Yunnan University. JH is a PhD student at Yunnan University.
